# Association of excessive mobile phone use during pregnancy with birth weight: an adjunct study in Kumamoto of Japan Environment and Children’s Study

**DOI:** 10.1186/s12199-017-0656-1

**Published:** 2017-06-08

**Authors:** Xi Lu, Masako Oda, Takashi Ohba, Hiroshi Mitsubuchi, Shota Masuda, Takahiko Katoh

**Affiliations:** 1 Department of Public Health, Faculty of Life Sciences, Kumamoto University, Kumamoto City, Kumamoto Japan; 2 The Southern Kyushu and Okinawa Unit Center, Faculty of Life Sciences, Kumamoto University, Kumamoto City, Kumamoto Japan; 3 Department of Obstetrics and Gynecology, Faculty of Life Sciences, Kumamoto University, Kumamoto City, Japan; 4 Department of Neonatology, Kumamoto University Hospital, Kumamoto City, Kumamoto Japan

## Abstract

**Background:**

Low birth weight has been shown to be closely associated with neonatal mortality and morbidity, inhibited growth, poor cognitive development, and chronic diseases later in life. Some studies have also shown that excessive mobile phone use in the postnatal period may lead to behavioral complications in the children during their growing years; however, the relationship between mobile phone use during pregnancy and neonatal birth weight is not clear. The aim of the present study was to determine the associations of excessive mobile phone use with neonatal birth weight and infant health status.

**Methods:**

A sample of 461 mother and child pairs participated in a survey on maternal characteristics, infant characteristics, and maternal mobile phone usage information during pregnancy.

**Results:**

Our results showed that pregnant women tend to excessively use mobile phones in Japan. The mean infant birth weight was lower in the excessive use group than in the ordinary use group, and the frequency of infant emergency transport was significantly higher in the excessive use group than in the ordinary use group.

**Conclusions:**

Excessive mobile phone use during pregnancy may be a risk factor for lower birth weight and a high rate of infant emergency transport.

## Background

Birth weight is the body weight of a baby at birth [1]. There is a widespread belief that the birth weight of babies has increased in the last several years [2, 3]. However, the Ministry of Health, Labour and Welfare [4] reported that the mean neonatal birth weight in Japan decreased from 3.24 kg for boys and 3.15 kg for girls in 1975 to 3.04 kg for boys and 2.96 kg for girls in 2014 and that the rate of low-birth-weight infants (birth weight below 2500 g) increased from 4.7% for boys and 5.5% for girls in 1975 to 8.5% for boys and 10.7% for girls in 2014. Therefore, the mean birth weight of Japanese infants is decreasing steadily, and previous studies support this finding [5, 6]. Low birth weight has been shown to be closely associated with neonatal mortality and morbidity, inhibited growth, poor cognitive development, and chronic diseases later in life [7]. A previous study reported that low birth weight predisposes individuals to chronic diseases, such as ischemic heart disease, diabetes, and hypertension, during adult life [8]. Maternal factors, such as primiparity, maternal smoking during pregnancy, maternal age, and multiple pregnancies, have been shown to be related with neonatal birth weight [8]; however, there is still no clarity on the relationship between many other factors and neonatal birth weight.

Recently, mobile phones have rapidly become important and widely available tools that are routinely used for a variety of purposes by a large number of people [9–12]. Most people have mobile phones and use them very often. Many people, especially the youth, use mobile phones to study, search for information on the Internet, play games, and communicate with others [13]. Some studies have shown that excessive mobile phone use may lead to a number of symptoms, such as headache, impaired concentration and memory, fatigue, and sleep disturbances [14, 15]. Additionally, excessive mobile phone use may negatively affect mental health and lead to depression, stress, and anxiety [9–14, 16].

Some studies reported that female individuals are prone to the adverse effects of excessive mobile phone use, and several studies have found that the levels of attachment to mobile phones and dependence on mobile phones are higher among female individuals than among male individuals [15, 17–21]. In a previous study of young adults (*n* = 1415), it was found that female individuals aged 20 years or older were nearly three times more likely than male individuals to agree with the statement, “I cannot imagine life without the mobile” (25 vs. 9%) [15]. However, the association between neonatal birth weight and mobile phone use during pregnancy remains unclear. A previous cohort study reported associations of prenatal and, to a lesser extent, postnatal exposures to mobile phones with behavioral problems in children aged 7 years [22]; however, very little is known about the correlation.

The present study aimed to determine the associations of excessive mobile phone use with neonatal birth weight and infant health status and to provide basic data about excessive mobile phone use during pregnancy in a local area of Japan.

## Methods

### Data collection

Data were obtained from the Japan Environment and Children’s Study (JECS) and JECS Adjunct Study in Kumamoto. In the JECS, three self-administered questionnaires and four medical record-transfer questionnaires were administered to the participants. Clinical measurements and biological samples were collected by trained nurses. The JECS included 103,106 expecting mothers, and the consent rate was 78.5%. The recruitment and exposure protocols are available elsewhere [23, 24]. The study in Kumamoto included 3012 mother-child pairs, and our study included data from only hospitals. Finally, we had 521 participants in our study.

In this study, we extracted basic data during pregnancy obtained with two maternal self-administered questionnaires and fetal data obtained with the first medical record-transfer questionnaire from JECS. Additionally, an original paper questionnaire in the JECS Adjunct Study was used to collect maternal mobile phone usage information during pregnancy (e.g., daily mobile phone use times, location of the phone during the day and at night, and power state (on/off) of the mobile phone during sleep). We distributed the questionnaire about daily mobile phone use when the expecting mothers were hospitalized before delivery or after delivery.

Igarashi et al. developed the Self-Perception of Text-Message Dependency Scale (STDS) for assessing text message dependency [25]. This self-reported scale measures the perception of people to their use of text messages and their attitudes toward compulsive text messaging in the context of interpersonal relationships. This scale consists of the following three subscales: emotional reaction, excessive use, and relationship maintenance. In the present study, a short version of the STDS was used. This version consists of 15 items with a 5-point scale (1 = “strongly disagree” and 5 = “strongly agree”). A higher score indicates greater dependency on text messaging and mobile phone use.

All expecting mothers from one rural area in Kumamoto, Japan, were considered for recruitment between 2012 and 2014. Recruitment was performed with the following two protocols: (1) recruitment at the time of the first prenatal examination at cooperating hospitals and (2) recruitment at local government offices.

### Research participants

We enrolled 521 participants and excluded (1) those who did not complete the first or second trimester questionnaires (MT1 or MT2) or the first medical record-transfer questionnaire (Dr-0M); (2) those who did not complete the mobile phone daily usage questionnaire; (3) those with missing data for the maternal MT1, MT2, Dr-0M, or mobile phone daily usage questionnaire; and (4) those who had multiple pregnancies. Finally, 461 mother and child pairs were included in our analysis.

### Outcome

Outcomes of this study were birth weight and infant health status (birth height, birth head circumference, birth chest circumference, mode of delivery, weeks of pregnancy, placental weight, low birth weight), infant emergency transport, and premature birth.

### Statistical analysis

The results are expressed as mean ± standard deviation (SD) or number (percentage). In our study, we used a cutoff of 15 points for the excessive use score in the STDS to determine excessive mobile phone use. Participants were divided into an ordinary use group (excessive use score ≤15 points) or excessive use group (excessive use score >15 points) [25]. For data analysis, we used the *t* test, *χ* ^2^ test, linear regression analysis, and logistic regression analysis. All statistical analyses were performed using SPSS (version 23.0; IBM Corp., Armonk, NY). A *P* value <0.05 was considered to indicate statistical significance.

## Results

The prevalence of excessive mobile phone use was 9.98% in our study. Table 1 shows the maternal characteristics and their differences between the ordinary and excessive use groups. Age at study entry was lower, the time of first use of a mobile phone was earlier, family income and education were lower, the frequencies of individuals who were unmarried and primiparous were higher, the sleeping time at night was later, the duration of playing games and using the mobile phone was longer, and the frequency of placing the mobile phone in the trouser or shirt pocket was higher in the excessive use group than in the ordinary use group (all *P* < 0.05).

**Table 1:** Maternal characteristics and their differences between the normal and excessive use groups

Maternal characteristics	Total (*N* = 461)	Mobile normal user (*N* = 415)	Mobile excessive user (*N* = 46)	*P* value
Maternal age	29.54 (±5.35)	30.03 (±5.08)	25.09 (±5.70)	*<0.001*
Mobile start age, years	17.29 (±2.81)	17.48 (±2.80)	15.63 (2.33)	*<0.001*
Wight change during pregnancy	11.25 (±3.88)	11.20 (±3.85)	11.67 (±4.12)	0.44
Maternal BMI before pregnancy	21.49 (±3.01)	21.52 (±3.43)	21.13 (±3.16)	0.45
Maternal smoking				
Yes	29 (6.3%)	24 (5.8%)	5 (10.9%)	0.19
No	432 (93.7%)	391 (94.2%)	41 (89.1%)	
Marital status				
Single	31 (6.7%)	18 (4.3%)	13 (28.3%)	*<0.001*
Married	430 (93.3%)	397 (95.7%)	33 (71.7%)	
Employment status				
Full-time job^a^	111 (24.1%)	96 (23.1%)	15 (32.6%)	0.09
Part-time job	152 (33.0%)	135 (32.5%)	17 (37.0%)	
Housewife	162 (35.1%)	152 (36.6%)	10 (21.7%)	
Independent business	18 (3.9%)	16 (3.9%)	2 (4.3%)	
No answers	18 (3.9%)	16 (3.9%)	2 (4.3%)	
Family income, ¥ per year				
<4,000,000	255 (55.3%)	225 (54.2%)	30 (65.2%)	*0.03*
4,000,000–7,999,999	141 (30.6%)	133 (32.0%)	8 (1.7%)	
8,000,000–11,999,999	15 (3.3%)	15 (3.6%)	0 (0%)	
≧12,000,000	12 (2.6%)	11 (2.7%)	1 (2.1%)	
No answer	38 (8.2%)	31 (7.5%)	7 (15.2%)	
Maternal schooling degree				
High school^a^	253 (54.9%)	216 (52.0%)	37 (80.4%)	*<0.001*
College^b^	206 (44.7%)	197 (47.5%)	9 (19.6%)	
Otherwise	2 (0.4%)	2 (0.5%)	0 (0%)	
Primiparity				
Yes	307 (66.6%)	281 (67.7%)	26 (56.5%)	*<0.001*
No	154 (33.4%)	134 (32.3%)	20 (43.5%)	
Time of laid up (excluding sleep), hours	5.00 (±3.68)	4.95 (±3.74)	5.57 (±2.93)	0.36
Time of watch TV, hours	2.67 (±2.05)	2.56 (±2.00)	3.59 (±2.27)	0.09
Time of playing game, minutes	28.0 (±56.5)	21.17 (±0.7)	89.90 (±1.8)	*<0.001*
Time of mobile use, minutes	133.7 (±191.4)	113.91 (±167.9)	314.91 (279.0)	*<0.001*
Designated spot for mobile				
Bag	364 (79.0%)	335 (80.7%)	29 (63.0%)	0.66
Trouser pocket	48 (10.4%)	34 (8.2%)	14 (30.4%)	
Shirt pocket	5 (1.1%)	5 (1.2%)	0 (0%)	
Coat pocket	17 (3.7%)	14 (3.4%)	3 (6.5%)	
Others	27 (5.9%)	27 (6.5%)	0 (0%)	
Designated spot for mobile				
Bag or coat pocket and others	408 (88.5%)	376 (90.6%)	32 (69.6%)	*<0.001*
Trouser pocket or shirt pocket	53 (11.5%)	39 (9.4%)	14 (30.4%)	
Bed time (time go to bed, PM)	10:54	10:54	11:48	*<0.001*
Power state of the mobile phone during sleep				0.65
On	425 (92.2%)	381 (91.6%)	44 (95.7%)	
Usually on	22 (4.8%)	20 (4.8%)	2 (4.3%)	
Sometimes on	6 (1.3%)	6 (1.4%)	0 (0%)	
Off	8 (1.7%)	8 (1.9%)	0 (0%)	
Location of the phone during sleep				0.33
0.5 m from abdominal	115 (24.9%)	99 (23.9%)	16 (34.8%)	
1 m from abdominal	236 (51.2%)	216 (52.0%)	20 (43.5%)	
1.5 m from abdominal	62 (13.3%)	55 (13.3%)	7 (15.2%)	
2 m from abdominal	48 (10.4%)	45 (10.8%)	3 (6.5%)	
Total sleep time (hours)	7.5 (±1.48)	7.5 (±1.46)	7.7 (±1.64)	0.35
Pregnancy complications				0.30
Yes	12 (2.6%)	12 (2.9%)	0 (0%)	
No	449 (97.4%)	403 (97.1%)	46 (100%)	
Obstetric labor complication				0.08
Yes	219 (47.6%)	193 (46.5%)	27 (58.7%)	
No	241 (52.4%)	222 (53.5%)	19 (41.3%)	

^a^Including junior high school

^b^Including technical college and junior college

Table 2 shows the infant characteristics and their differences between the ordinary and excessive use groups. The mean birth weight and birth chest circumference were higher in the ordinary use group than in the excessive use group (3167.16 ± 394.05 g vs. 3037.37 ± 324.87 g, *P* < 0.05, and 32.45 ± 1.65 cm vs. 31.94 ± 1.77 cm, *P* < 0.05, respectively). The frequency of emergency transportation was higher in the excessive use group than in the ordinary use group (*P* < 0.05). However, the proportion of low-birth-weight infants was not significantly different between the two groups.

**Table 2:** Infant characteristics and their differences between the normal and excessive use groups

Infant characteristics	Total (*N* = 461)	Mobile ordinary user (*N* = 415)	Mobile excessive user (*N* = 46)	*P* value
Gender				
Boy	247 (53.6%)	227 (54.7%)	20 (43.5%)	0.15
Girl	214 (46.4%)	188 (45.3%)	26 (56.5%)	
Mode of delivery				
Transvaginal	386 (83.7%)	346 (83.4%)	40 (87.0%)	0.53
Caesarean section	75 (16.3%)	69 (16.6%)	6 (13.0%)	
Gestational age	39.39 (±1.18)	39.41 (±1.18)	39.21 (±1.18)	0.27
Birth weight (g)	3154.25 (±389.34)	3167.16 (±394.05)	3037.74 (±324.87)	*0.03*
Birth height (cm)	48.94 (±1.70)	48.93 (±1.72)	49.00 (±1.51)	0.792
Birth head circumference (cm)	33.35 (±1.38)	33.39 (±1.37)	32.98 (±1.43)	0.06
Birth chest circumference (cm)	32.40 (±1.67)	32.45 (±1.65)	31.94 (±1.77)	*0.05*
Placental weight (g)	587.74 (±111.76)	588.71 (±114.11)	579.00 (88.34)	0.50
Low birth weight				
1500–2500 g	16 (3.5%)	15 (3.6%)	1 (2.2%)	0.61
>2500 g	445 (96.5%)	400 (96.4%)	45 (97.8%)	
Premature birth				
Yes	14 (3.0%)	12 (2.9%)	2 (4.3%)	0.59
No	447 (97.0%)	403 (91.7%)	44 (95.7%)	
Emergency transport				
Yes	10 (2.2%)	7 (1.7%)	3 (6.5%)	*0.03*
No	451 (97.8%)	408 (98.3%)	43 (93.5%)	

On assessing the correlation between predictor variables and body weight, we found that maternal age, maternal body mass index before pregnancy, birth height, birth head circumference, birth chest circumference, and placental weight were positively correlated with birth weight. With regard to sex differences, the birth weight was lower in female infants than in male infants. However, maternal smoking history and excessive mobile phone use were not significantly correlated with birth weight. Additionally, primiparity and the designated location for the mobile phone were not significantly associated with birth weight. The correlation coefficient in univariate analysis in terms of the association of excessive mobile phone use with birth weight was −0.10 (*P* < 0.05).

We performed a linear regression analysis to identify the predictors of gestational age, birth weight, and low birth weight. The variables that showed statistical significance are presented in Table 3. Birth weight, birth chest circumference, and infant sex showed positive correlation with gestational age, while placental weight and primiparity had negative correlation. Birth chest circumference, birth height, placental weight, and maternal body mass index before pregnancy showed positive correlation with birth weight, while excessive mobile phone use showed negative correlation. Maternal age, birth height, maternal BMI before pregnancy, birth head circumference, primiparity, maternal smoking, excessive mobile phone use, maternal complications, and obstetric labor complications were predictors added in the analysis; however, they were deleted during analysis. There were only 16 low-birth-weight infants in our study. After linear regression analysis, only the chest circumference at birth had positive correlation with low birth weight, and other predictors were non-significant during analysis.

**Table 3:** Association between predictor variables and birth weight

Predictor variables	Gestational age^a^ (*N* = 416)	Birth weight^b^ (*N* = 416)	Low birth weight (<2500 g)^c^ (*N* = 16)
	B (95%CI)	B (95%CI)	B (95%CI)
	31.55 (29.42~33.68)**	−6401.58 (−6943.38~−5859.78)**	−1198.61 (−2499.57~102.34)**
Birth weight	0.001 (0.001~0.002)**	–	–
Birth chest circumference	0.15 (0.06~0.25)**	112.25 (100.75~123.75)**	121.08 (75.95~166.22)**
Birth height	–	89.48 (78.90~100.07)**	–
Placental weight	−0.003 (−0.004~−0.002)**	0.60 (0.45~0.75)**	–
Maternal BMI before pregnancy	–	5.85 (1.67~10.03)**	–
Primiparity	−0.38 (−0.57~0.20)**		
Infant sex	0.26 (0.07~0.44)**	–	–
Gestational age	–	27.09 (13.02~41.16)**	–
Mobile excessive use	–	−66.46 (−114.46~−18.46)**	–

** *P* < 0.01

^a^ *R* ^2^ = 0.34, ANOVA *P* < 0.01. Predictor variables deleted during analysis: maternal age, birth height, maternal BMI before pregnancy, maternal age, birth head circumference, primiparity, maternal smoking, mobile excessive use, maternal complications, and obstetric labor complication

^b^ *R* ^2^ = 0.85, ANOVA *P* < 0.01. Predictor variables deleted during analysis: maternal age, birth head circumference, primiparity, infant sex, maternal smoking, maternal complications, and obstetric labor complication

^c^ *R* ^2^ = 0.70, ANOVA *P* < 0.001. Predictor variables deleted during analysis: maternal age, birth height, birth head circumference, placental weight, maternal BMI before pregnancy, primiparity, infant sex, gestational age, mobile excessive use, maternal smoking, maternal complications, and obstetric labor complication

We assessed emergency transport and premature birth between the ordinary and excessive use groups (Table 4). The risk of emergency transport was significantly higher in the excessive use group than in the ordinary use group (crude OR = 4.07, 95%CI = 1.01–16.30; adjusted OR = 7.93, 95%CI = 1.40–44.85). The risk of premature birth was not significantly different between the ordinary and excessive use groups.

**Table 4:** Infant emergency transport and premature birth differences between the normal and excessive use groups

	Infant emergency transport		Premature birth	
	Crude OR (95%CI)	Adjusted OR^a^ (95%CI)	Crude OR (95%CI)	Adjusted OR^a^ (95%CI)
Normal user	1	1	1	1
Excessive user	4.07 (1.01–16.30)*	7.93 (1.40–44.85)*	0.66 (0.14–3.02)	0.67 (0.09–4.97)

*P < 0.05

^a^Adjust for birth weight, birth height, birth head circumference, birth chest circumference, placental weight, maternal age, primiparity, maternal smoking, and maternal BMI before pregnancy

## Discussion

To our knowledge, this is the first study on the prevalence of excessive mobile phone use during pregnancy in Japanese women from a local area. Our study findings suggest that excessive mobile phone use in Japan is not limited to students and can be noted in adult women, even during pregnancy. The prevalence of excessive mobile phone use was 9.98%, which was higher than that reported among women employed in companies (5.4%) [16].

With regard to maternal characteristics, the maternal age and age at first use of a mobile phone were lower in the excessive use group than in the ordinary use group. These findings are consistent with the results of previous studies [9, 16]. Young individuals tend to show high excitement and interest in the use of a new mobile phone. We also found that single mothers had excessive mobile phone use. The frequencies of loneliness, depression, and anxiety may be higher among single mothers than among mothers who have support during pregnancy. In a previous study, Shaw and Grant found that Internet chat alleviated loneliness and depression and increased perceived social support and self-esteem [26]. Additionally, Igarashi and Yoshida [27] found that among first-year university students, a higher frequency of text messaging at the beginning of the first semester was correlated with a lower feeling of loneliness at the end of the semester. We believe that single mothers need more time to use a mobile phone as a communication tool to maintain contact with others. We found that the family income and maternal schooling degree were lower in the excessive use group than in the ordinary use group; however, we did not identify any previous study to support these results. Nonetheless, we believe that different income and education levels may influence mobile phone use frequency and habit. We also found that the excessive use group preferred to place the mobile phone in the trouser or shirt pocket, indicating that the excessive use group preferred to have the mobile phone in an easily accessible location.

With regard to infant characteristics, birth weight and chest circumference at birth were lower and the emergency transport rate was higher in the excessive use group than in the ordinary use group. In the linear regression analysis, excessive mobile phone use was a significant predictor of low birth weight. In the logistic regression analysis, interestingly, we found that excessive mobile phone use increased the emergency transport rate, even after adjusting for confounding factors. Many factors affect the duration of gestation and fetal growth and, thus, the birth weight. They relate to the infant, mother, or physical environment and play important roles in determining birth weight and the future health of the infant. Our results showed that excessive mobile phone use during pregnancy may decrease infant birth weight, although this does not result in low-birth-weight infants. With regard to the mechanism, it has been shown that the levels of depression and state-trait anxiety are higher, and sleep quality is poorer in users who showed texting and digital audio player dependence than in ordinary users [28]. Another study reported that anxiety and dependence increased with daily smartphone usage and a high rate of nighttime awakening was noted, which, in turn, affected sleep [29]. Our study suggests that excessive mobile phone use during pregnancy may cause mental problems, such as anxiety and depression, and health problems, such as sleep problems and sleeplessness. We also found that the sleep time was later in the excessive use group than in the ordinary use group, and therefore, excessive users may have poor sleep quality; however, there was no significant difference in the total sleep duration between the excessive and ordinary use groups. The maternal health and mental problems may lead to a low birth weight and neonatal health, which may eventually necessitate infant emergency transport.

### Limitations

The present study had some limitations. First, the sample size was relatively small, and therefore, it may not provide sufficient power to estimate the association between excessive mobile phone use and infant birth weight. The number of emergency transports was only 10, and there was a possibility of chance with regard to our finding. Second, we did not account for the potential effects of mental health, such as depression and anxiety, which were mainly noted in excessive mobile phone users and users with high dependency and may be predictors of birth weight. Third, all information was reported by the participants themselves; therefore, the reliability of the responses may not be high. Despite these limitations, our study had some merits and values. Finally, the subjects were not representative of all pregnant Japanese women, as they were recruited from a local area of Japan. Therefore, we might need a larger sample from all areas of Japan to verify our results. Although our research is important, there are many shortcomings. To our knowledge, this is the first study to analyze the prevalence of excessive mobile phone use during pregnancy in a local area in Japan.

## Conclusions

Pregnant women tend to excessively use mobile phones in Japan. Excessive mobile phone use during pregnancy may be a risk factor for lower birth weight and a high rate of infant emergency transport.

## Abbreviations

JECS: Japan Environment and Children’s Study
SD: Standard deviation
STDS: Self-Perception of Text-Message Dependency Scale
